# Exercise-Driven Comprehensive Recovery: Pulmonary Rehabilitation’s Impact on Lung Function, Mechanics, and Immune Response in Post-COVID-19 Patients

**DOI:** 10.3390/idr17010001

**Published:** 2025-01-03

**Authors:** Maysa Alves Rodrigues Brandao-Rangel, Boris Brill, Guilherme Eustáquio Furtado, Catharine Cássia Lanna Freitas-Rolim, Anamei Silva-Reis, Victor Hugo Souza-Palmeira, Renilson Moraes-Ferreira, Vanessa Lopes-Silva, Regiane Albertini, Wendel Simões Fernandes, Sérgio César Ferreira, Ricardo César Alves Ferreira, Jose Roberto Mateus-Silva, Carlos Rocha Oliveira, Claudio Ricardo Frison, Rodolfo P. Vieira

**Affiliations:** 1 Postgraduate Program in Sciences of Human Movement and Rehabilitation, Federal University of São Paulo (UNIFESP), Santos 11060-001, Brazil; maysarangel_4@hotmail.com (M.A.R.B.-R.); victorsp07@outlook.com (V.H.S.-P.); renilsonmoraesferreira@gmail.com (R.M.-F.); vanessalopessilva15@gmail.com (V.L.-S.); regiane.albertini@unifesp.br (R.A.); wen_sfernandes@hotmail.com (W.S.F.); sergiocesarferreira@gmail.com (S.C.F.); ricardocalves@hotmail.com (R.C.A.F.); claudiofrison@gmail.com (C.R.F.); 2 Department of Emergency Medicine, Leniado Medical Center, Divrei Khayim St. 16, Nethanya 4244916, Israel; drborisbrill@gmail.com; 3 Polytechnic Institute of Coimbra, Applied Research Institute, Rua da Misericórdia, Lagar dos Cortiços–S, Martinho do Bispo, 3045-093 Coimbra, Portugal; guilherme.furtado@ipc.pt; 4 Research Centre for Natural Resources Environment and Society (CERNAS), Polytechnic Institute of Coimbra, Bencanta, 3045-601 Coimbra, Portugal; 5 Laboratory of Pulmonary and Exercise Immunology, Evangelical University of Goias (UniEvangélica), Avenida Universitária Km 3,5, Anápolis 75083-515, Brazil; catharinefreitas@gmail.com (C.C.L.F.-R.); anameisreis97@gmail.com (A.S.-R.); 6 Department of Research and Development, GAP Laboratory of Biotechnology, Sao Jose dos Campos 12243-020, Brazil; jrm@gap.com.br (J.R.M.-S.); pharmacologia@hotmail.com (C.R.O.); 7 Department of Research and Development, Brazilian Institute of Teaching and Research in Pulmonary and Exercise Immunology (IBEPIPE), Rua Pedro Ernesto 240, Sao Jose dos Campos 12245-520, Brazil

**Keywords:** post-COVID syndrome, respiratory recovery, inflammation markers, immune modulation, exercise-based therapy

## Abstract

**Introduction:** We sought to evaluate the effects of a 12-week pulmonary rehabilitation (PR) program on lung function, mechanics, as well as pulmonary and systemic inflammation in a cohort of 33 individuals with moderate to severe post-COVID-19. **Material and Methods**: The pulmonary rehabilitation (PR) program employed a combination of aerobic and resistance exercises. Thirty minutes of treadmill training at 75% of the maximum heart rate, combined with 30 min resistance training consisting of 75% of one maximum repetition, three times a week throughout 12 weeks. **Results**: PR improved the lung function, FVC (*p* < 0.02), FEV1 (*p* < 0.02), FEV1/FVC (*p* < 0.01), MEF25% (*p* < 0.006), MEF50% (*p* < 0.03), and MEF75% (*p* < 0.02). PR also positively influenced lung mechanics, reducing respiratory impedance (Z5Hz, *p* < 0.03), respiratory reactance (X5Hz, *p* < 0.01), resistance of the entire respiratory system (R5Hz, *p* < 0.03), central airway resistance (RCentral, *p* < 0.03), and peripheral airway resistance (RPeripheral, *p* < 0.02). Moreover, muscle strength gains were evident, with significant improvements observed in hand grip strength for both the right (*p* < 0.02) and left (*p* < 0.01) hands, as well as maximal inspiratory (*p* < 0.02) and expiratory (*p* < 0.03) pressures. Additionally, PR exhibited anti-inflammatory effects by reducing the pro-inflammatory cytokines IL-1β (*p* < 0.0001) and IL-6 (*p* < 0.0001) and increasing the anti-inflammatory IL-1RA (*p* < 0.0004) and IL-10 (*p* < 0.003) and anti-viral IFN-γ (*p* < 0.0002) and IFN-β (*p* < 0.008) cytokines in breath condensate and serum samples. **Conclusions**: Collectively, these findings highlight the effectiveness of PR in ameliorating COVID-19 sequel across respiratory system, skeletal muscle, and immune responses. This highlights its promising potential as a therapeutic intervention for individuals recovering from COVID-19.

## 1. Introduction

The highly infectious and pathogenic novel coronavirus (SARS-CoV-2) emerged, causing a global pandemic with a wide range of symptoms, including mild to severe cases and even fatalities. To date, more than 767 million individuals have been infected, resulting in over 6.9 million deaths worldwide [1]. While COVID-19 primarily impacts the respiratory system, emerging evidence indicates its effects on various organs and systems [2]. Despite substantial knowledge about acute sequel on lung function [3], the long-term consequences of COVID-19 remain incompletely understood, although the literature already presents some prejudice for lung function, quality of life, and effort tolerance [4]. In fact, in this study performed on an Indian population, regarding the lung function, the authors demonstrated a reduction in total lung capacity (TLC), in the VEF1/FVC, and a diminished lung diffusion capacity (DLCO) [4]. Notably, severe cases of the disease often lead to significant dysregulation of the immune system, resulting in sequel that affect multiple organs and systems [5].

Furthermore, genetic defects in interferon (IFN) generation or the presence of autoantibodies that disrupt IFN function are clear risk factors for severe COVID-19 [6]. SARS-CoV-2 infects alveolar macrophages in the lungs, prompting them to release Th1 chemoattractants. Consequently, recruited and activated Th1 cells produce IFN-γ, further enhancing the antiviral and pro-inflammatory functions of resident macrophages mobilized by monocytes [6]. Conversely, excessive suppression of Th1 and IFN-γ production is associated with heightened production of IL-2, IL-6, and TNF-α [6]. These conditions, characterized by low levels of IFN-γ in the bloodstream, are linked to the severity of COVID-19 and post-COVID-19 syndrome [7]. In addition, the importance of cytokines released into the lungs, as measured in the breath condensate, are indicative of their central role in the pathogenesis and severity of post-COVID-19 [8].

Persistent symptoms of the post-COVID-19 syndrome encompass a range of issues, including fatigue, muscle weakness, sleep disturbances, anxiety, depression, difficulty concentrating, muscle and joint pain, and autonomic dysfunction, among others [9,10,11,12]. These symptoms affect approximately 20% of patients and can persist for six months or more after the acute phase of COVID-19 infection [9,10,11,12].

Given this scenario, there is a compelling need for rehabilitative interventions aimed at preventing or mitigating the severity of post-COVID-19 syndrome. Previous studies have demonstrated the effectiveness of pulmonary rehabilitation programs (PRPs) primarily involving aerobic and resistance exercises in improving lung function among patients with COPD [13] and asthma [14]. Additionally, these exercises have shown potential in reducing systemic inflammation in asthma [14] and COPD patients [15], as well as improving both pulmonary and systemic inflammation in obese women [16]. Furthermore, aerobic and resistance exercises have been proven to enhance lung mechanics in these populations [14,16].

Therefore, this study aims to investigate whether a 12-week pulmonary rehabilitation (PR) program, encompassing aerobic and resistance training, may improve lung function, mechanics, peripheral and respiratory muscle strength, as well as pulmonary and systemic immune responses in 33 moderate and severe post-COVID-19 patients.

## 2. Material and Methods

### 2.1. Study Design and Participants

In this clinical study, a control group consisting of post-COVID-19 patients without rehabilitation was not included due to limitations imposed by the COVID-19 pandemic and ethical considerations outlined in the Helsinki treaty. The study was approved by the Ethics Committee of Anhembi Morumbi University (registration number 4.637.661). The study protocol is registered under number 45169821.5.0000.5492 from the Brazil Platform (http://plataformabrasil.saude.gov.br/ (accessed on 21 May 2024)). Numerous volunteers seeking rehabilitation expressed concerns and complaints that made the establishment of a control group impractical. Therefore, this study was conducted between January and December 2021, a period marked by significant challenges due to the ongoing pandemic. Initially, 104 patients with moderate and severe COVID-19 symptoms were enrolled. However, it is worth noting that only 33 patients with these severity levels ultimately accepted to participate in the study and completed the study. Additionally, several post-COVID-19 patients expressed a preference for undergoing pulmonary evaluations without participating in the pulmonary rehabilitation program. Their choice was often driven by concerns about the potential risk of contracting a new COVID-19 infection.

### 2.2. Participant Selection Criteria

All participants received a COVID-19 diagnosis through RT-PCR testing, adhering to the manufacturer’s recommended criteria. They were referred to our study by family physicians upon their discharge from the hospital. The study was conducted at the Laboratory of Pulmonary Immunology and Exercise (LABPEI), Federal University of São Paulo (UNIFESP). In addition, the present study enrolled volunteers with post-COVID-19 syndrome without any restriction related to race, gender, religion, or socio-economic status.

### 2.3. Inclusion Criteria

Participants eligible for this study met the following criteria: A diagnosis of COVID-19 confirmed by RT-PCR testing and presentation of moderate and severe symptoms, in accordance with the WHO classification for the year 2021 [16].

### 2.4. Exclusion Criteria

Participants meeting any of the following criteria were excluded from the study: the presence of viral infections caused by etiologic agents other than SARS-CoV-2, a diagnosis of other respiratory tract diseases with a negative COVID-19 test result, musculoskeletal limitations that hindered participation in the study, a oxygen saturation level below 90%, and cognitive limitations that impeded the understanding of exercise instructions and commands.

### 2.5. Evaluation of Inflammatory Mediators in Breath Condensate

Pulmonary inflammation has a key role in pathogenesis and progression and severity of COVID-19 [8]. Consequently, the use of measurement of cytokines in breath condensate has emerged as a non-invasive, sensitive, and very useful tool to assess the humoral response from the lungs [8,17]. To collect exhaled breath condensate, we utilized the RT-Tube from Respiratory Research (USA) [14,16]. Patients were instructed to maintain their tidal volume for a duration of 10–15 min [14,16]. The collected samples were promptly preserved at −86 °C to ensure the stability of inflammatory mediators. The analysis encompassed a range of inflammatory mediators, including IL-10 (DY217B), IL-1ra (DY280), IL-1beta (DY201), IL-2 (DY202), IL-6 (DY206), TNF-alpha (DY210), IFN-gamma (INF-y) (DY217B), and IFN-beta (IFN-β) (DY285B) [14,16]. These analyses were conducted using the ELISA technique, with commercial kits sourced from R&D Systems^®^ (Minneapolis, MN, USA). The procedures strictly adhered to established technical protocols. Readings and measurements were performed using the SpectraMax i3 equipment (Molecular Devices^®^, San Jose, CA, USA) [14,16]. This approach allowed for a comprehensive assessment of the levels of these essential inflammatory markers within the breath condensate samples.

### 2.6. Cellular and Humoral Immune Response Analysis

Venous blood was collected into tubes containing EDTA K3 anticoagulant. A precise volume of 25 microliters of blood was used for blood analysis (red and white cells) using the Sysmex XS-800i equipment [14,16]. The remaining blood volume was subjected to centrifugation at 1800× *g* for 7 min at 4 °C. Subsequently, the resulting plasma was carefully stored at −86 °C for the measurement of various inflammatory mediators, including IL-10 (DY217B), IL-1ra (DY280), IL-1beta (DY201), IL-2 (DY202), IL-6 (DY206), TNF-alpha (DY210), IFN-gamma (INF-y) (DY217B), and IFN-beta (IFN-β) (DY285B). These measurements were carried out using the ELISA technique with commercial kits sourced from R&D Systems (USA), following established protocols [14,16]. The readings and measurements were conducted using the SpectraMax i3 equipment (Molecular Devices, CA, USA). This comprehensive approach allowed us to assess the levels of these critical inflammatory markers in the plasma samples accurately.

### 2.7. Body Composition Characterization

We collected anthropometric measurements, which included weight and height. Additionally, body composition was evaluated using Bioimpedance analysis conducted with the Maltron 920-II-S equipment from Maltron Inc^®^., Rayleigh, Essex, UK [14,16].

### 2.8. Hand Grip Strength Assessment

Grip strength was measured using a hand dynamometer (Jamar^®^, Sammons Preston Rolyan, Bolingbrook, IL, USA) equipped with a hydraulic system featuring two parallel handles [14]. The dynamometer records the strength of a maximal contraction and reports it in kilograms. During the assessment, participants were instructed to use their dominant limb while sitting with their elbow flexed at 90 degrees and maintaining the forearm and wrist in a neutral position. Five measurements were taken, and the highest value was assumed as the hand grip strength.

### 2.9. Respiratory Muscle Strength Assessment

The assessment of respiratory muscle strength was conducted in accordance with the guidelines established by the American Thoracic Society/European Respiratory Society (ATS/ERS) [14,16,18]. For this purpose, we utilized a Globalmed compound gauge (MVD-300 V.1.1 Microhard System Globalmed, Porto Alegre, Brazil) with a pressure range of 0 to 120 cmH_2_O. Participants were instructed to sit with an upright trunk posture and perform two practice maneuvers to become familiar with the process. To measure maximum inspiratory pressure (MIP), participants initiated the maneuver with a maximal expiration (expiratory reserve volume—ERV) and followed it with a maximal inspiration [14,16,18]. For the maximum expiratory pressure (MEP) measurement, the starting point was the total lung capacity (TLC), and participants then performed a maximal expiration [14,16,18]. To qualify as a valid measurement, participants had to maintain the reached position at the end of maximal efforts for at least one second. A total of five maneuvers were performed, with at least three deemed acceptable, and a minimum of two of these had to be reproducible. A one-minute rest interval was observed between each maneuver [14,16,18].

### 2.10. Fractional Exhaled Nitric Oxide

The second analysis conducted was the levels of fractional exhaled nitric oxide (FeNO), which were measured using the portable nitric oxide device NOBreath (Bedfont Scientific, Kent, UK) to evaluate pulmonary inflammation [14,16,19].

### 2.11. The Neutrophil-to-Lymphocyte Ratio

The neutrophil-to-lymphocyte ratio (NLR) is a biomarker of inflammation that serves as an indicator of systemic inflammation. It is defined by the absolute number of neutrophils divided by the absolute number of lymphocytes. This ratio provides a simple and cost-effective measure and can be derived from routine blood count laboratory examinations performed in hospitals. The NLR has been extensively studied as a prognostic indicator for various diseases, including cancer, community-acquired pneumonia, and sepsis [20]. It offers valuable insights into the inflammatory status of individuals and can aid in assessing the severity and prognosis of these conditions.

### 2.12. Evaluation of Lung Function and Mechanics

Lung function assessment employed the spirometry method, while pulmonary mechanics were appraised using the impulse oscillometry system method (IOS Masterscreen, Jaeger, Germany). These assessments were performed both before and after the administration of a bronchodilator (Salbutamol sulfate 400 mcg), following established guidelines from the American Thoracic Society [14,16,21]. The clinical parameters encompassed forced vital capacity—predicted (FVC%); forced expiratory volume in 1 s—predicted (FEV1%); FEV1/FVC%; forced expiratory volume in 3 s (FEV3); forced expiratory volume in 6 s (FEV6); peak expiratory flow—predicted (PEF%); maximal expiratory flow at 25%—predicted (MEF25%); MEF50%; MEF75%; and MEF25/75%, as well as various pulmonary mechanics parameters: respiratory system impedance—predicted (Z5Hz%); total respiratory system resistance—predicted (R5Hz%); proximal airway resistance—predicted (R20Hz%); distal airway resistance (R5Hz-R20Hz); lung reactance (X5Hz%); central resistance (RCentral); peripheral resistance (RPeripheral); and resonance frequency (RFres) [14,16,21].

## 3. Pulmonary Rehabilitation Training Protocol: Aerobic Training and Resistance Training

### 3.1. Aerobic Training

Aerobic training sessions were conducted on a treadmill three times per week. The intensity of these sessions was maintained at a moderate level, targeting a range of 70% to 80% of the maximal heart rate. Each aerobic training session had a duration of 30 min. This training regimen was consistently followed for a total period of 12 weeks, as outlined in the study by Matsubara et al. (2014) [16,22].

### 3.2. Resistance Training

Resistance training comprised five exercises: deadlift, incline bench press, seated row, abdominal exercises, and dumbbell shoulder press [14]. The entire training session took approximately 30 min to complete. The first week of the training program was dedicated to familiarizing the participants with these exercises. Over the course of 12 weeks, three training sessions were conducted per week. Each exercise consisted of 8 to 12 repetitions per set, with three sets performed for each exercise. Rest intervals of 1 to 2 min were observed between sets [16]. The training load was progressively increased on a weekly basis, ranging from 2% to 10% increments of the initial load, whenever participants could successfully perform the entire range of motion (maximal amplitude) for two consecutive training sessions [14]. Additionally, signs of fatigue, such as a tendency to fail during the concentric phase of the movement, reduced velocity, apnea, and isometric contraction period, were monitored based on criteria outlined by Garber et al. (2011) [16,23].

The evaluations were conducted in a specific sequence both before and after the 12-week training period. The post-training evaluation occurred 24 h after the last exercise session and consistently took place between 8:00 a.m. and 12:00 p.m.

### 3.3. Statistical Analysis

The data were analyzed using GraphPad Prism 8.0.1 software (San Diego, CA, USA). Data descriptions were compiled as mean ± standard deviation (M ± SD), median (interquartile 1st and 3rd), and difference in means (DM) and confidence interval at the 95% level (95% CI). The distribution of data normality was assessed using the Shapiro–Wilk test. The Student’s t test was used to compare data with normal distribution. One-way analysis of variance (ANOVA) (Bonferroni’s) was used for parameters of lung function and mechanics.

## 4. Results

### 4.1. Effects of Pulmonary Rehabilitation on Clinical Features of Post-COVID-19 Patients

As illustrated in Table 1, the results unequivocally showcase the substantial impact of the pulmonary rehabilitation protocol implemented in this study. Notably, there were significant reductions observed in body weight (*p* < 0.0002), BMI (*p* < 0.0002), waist circumference (*p* < 0.0004), and fat mass (*p* < 0.0001). Simultaneously, a noteworthy increase was observed in fat-free mass (*p* < 0.0002). Additionally, several physiological parameters exhibited favorable changes, including resting heart rate (*p* < 0.0001), systolic (*p* < 0.0002) and diastolic (*p* < 0.0002) blood pressure, SpO_2_% (*p* < 0.0001), and FeNO (*p* < 0.0001), which demonstrated reductions. Furthermore, hand grip strength (*p* < 0.0005), MIP (*p* < 0.0001), and MEP (*p* < 0.0001) all exhibited significant increases. Before the start of the pulmonary rehabilitation program, 78% of the participants had handgrip strength values below those expected for their age group and gender, with a mean of 20 Kgf compared to the normative value of 35 kgf [14]. After the program, this proportion decreased to 37%, with a significant increase in the average to 38 Kgf (*p* < 0.0005). Regarding the MIP and MEP, 82% of the participants initially presented values below the normative references for MIP and MEP. After rehabilitation, there was a significant reduction in the proportion of patients outside the normative range, from 82% to 38% for MIP and from 78% to 29% for MEP. The mean MIP increased from 40 cmH_2_O to 90 cmH_2_O (*p* < 0.0001), while the mean MEP improved from 30 cmH_2_O to 95 cmH_2_O (*p* < 0.0001). These findings unequivocally highlight the beneficial effects of the pulmonary rehabilitation protocol implemented in this research. Additionally, the results indicate that the pulmonary rehabilitation protocol resulted in a decrease in the counts of basophils (*p* < 0.0089), monocytes (*p* < 0.0026), and neutrophils (*p* < 0.0004), accompanied by a significant reduction in the neutrophil/lymphocyte ratio (*p* < 0.0001). These observations provide strong evidence supporting the presence of a robust systemic anti-inflammatory effect associated with pulmonary rehabilitation in post-COVID-19 patients.

### 4.2. Pulmonary Rehabilitation Reduces Inflammatory and Increases Anti-Inflammatory Mediators in Breath Condensate

**Figure 1:**
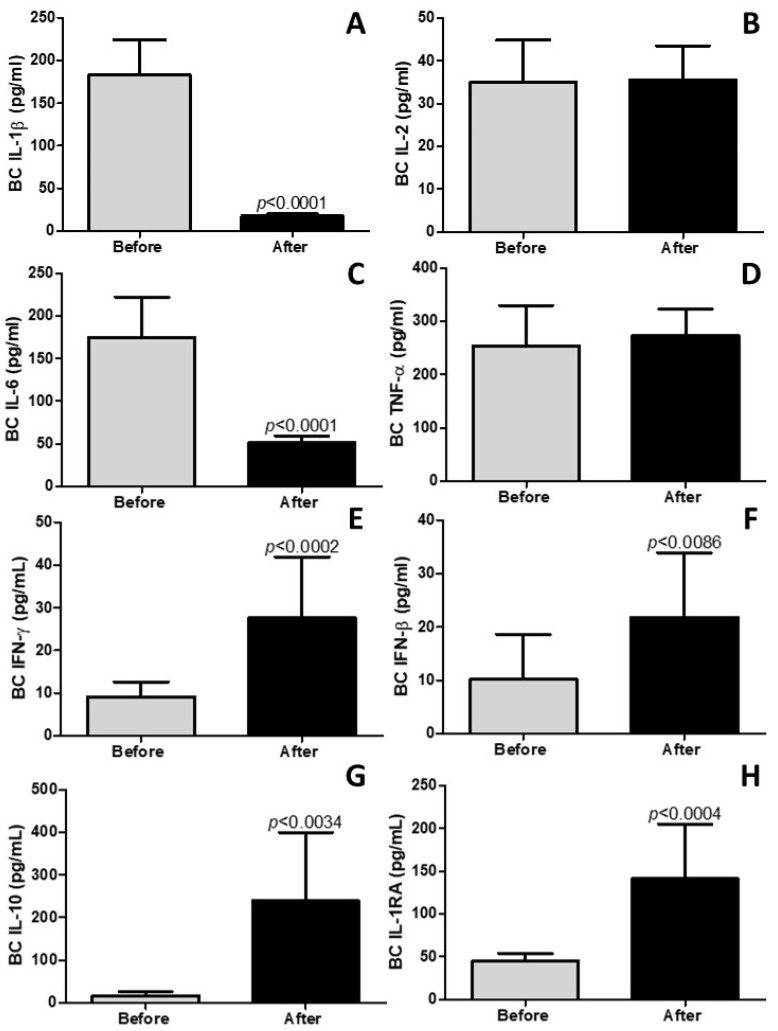
Cytokine levels in lungs measured in breath condensate (BC). “IL” stands for interleukin, “TNF-α” represents tumor necrosis factor alpha, “IFN” denotes interferon, and “IL-1RA” signifies interleukin 1 receptor antagonist. The levels of the following cytokines in BC are presented in (**A**) IL-1β, (**B**) IL-2, (**C**) IL-6, (**D**) TNF-α, (**E**) IFN-γ, (**F**) IFN-β, (**G**) IL-10, (**H**) IL-1RA. The results are expressed in picograms per milliliter (pg/mL).

The results depicted in Figure 1 highlight a significant pulmonary anti-inflammatory effect resulting from pulmonary rehabilitation. Specifically, in Figure 1A,C, it is evident that pulmonary rehabilitation led to a noteworthy reduction in the pulmonary levels of the pro-inflammatory cytokines IL-1β (*p* < 0.0001) and IL-6 (*p* < 0.0001), respectively. Conversely, Figure 1B,D reveal no discernible impact of pulmonary rehabilitation on the pulmonary levels of IL-2 and TNF-α, respectively (*p* > 0.05). Furthermore, Figure 1E,F illustrate that pulmonary rehabilitation induced an increase in the pulmonary levels of the anti-viral cytokines IFN-γ (*p* < 0.0002) and IFN-β (*p* < 0.0086). Additionally, Figure 1G,H demonstrate a notable rise in the pulmonary levels of the anti-inflammatory cytokines IL-10 (*p* < 0.0034) and IL-1RA (*p* < 0.0004) as a result of pulmonary rehabilitation. These findings collectively emphasize the substantial pulmonary anti-inflammatory effects associated with the pulmonary rehabilitation regimen employed in this study.

### 4.3. Pulmonary Rehabilitation Reduces Inflammatory and Increases Anti-Inflammatory Mediators in Blood (Serum)

**Figure 2:**
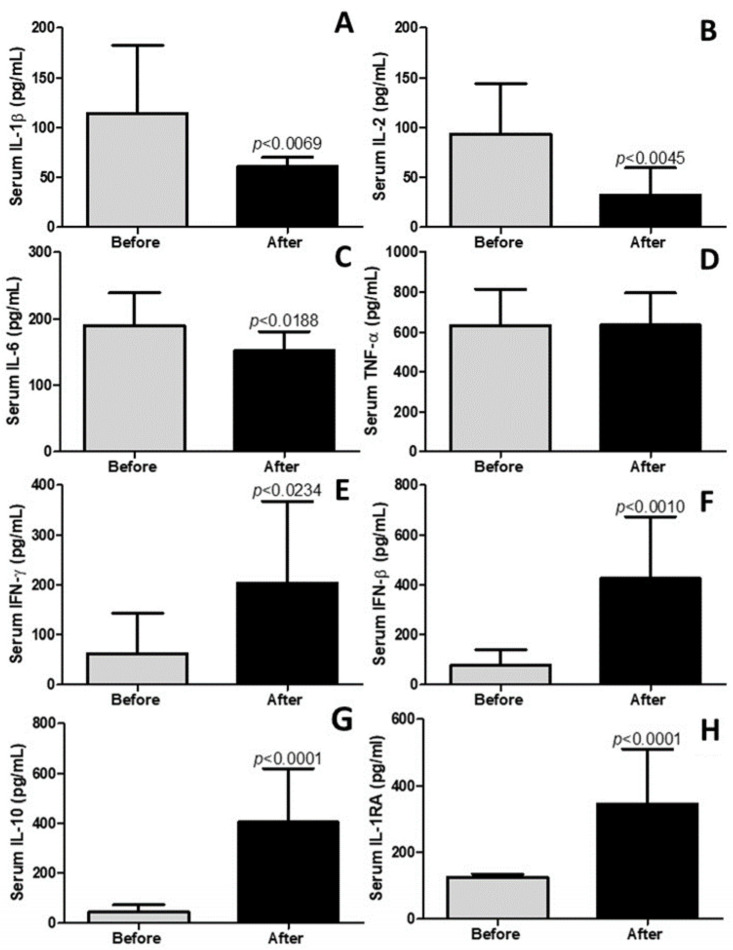
Cytokine levels in the blood measured in serum. “IL” stands for interleukin, “TNF-α” represents tumor necrosis factor alpha, “IFN” denotes interferon, and “IL-1RA” signifies interleukin 1 receptor antagonist. The levels of the following cytokines in BC are presented in (**A**) IL-1β, (**B**) IL-2, (**C**) IL-6, (**D**) TNF-α, (**E**) IFN-γ, (**F**) IFN-β, (**G**) IL-10, (**H**) IL-1RA. The results are expressed in picograms per milliliter (pg/mL).

The results presented in Figure 2 underscore a significant systemic anti-inflammatory effect arising from pulmonary rehabilitation. Specifically, as depicted in Figure 2A–C, it is evident that pulmonary rehabilitation led to a notable reduction in the pulmonary levels of the pro-inflammatory cytokines IL-1β (*p* < 0.0059), IL-2 (*p* < 0.0045), and IL-6 (*p* < 0.0188), respectively. Conversely, Figure 2D reveals no discernible impact of pulmonary rehabilitation on the pulmonary levels of TNF-α (*p* > 0.05). Moreover, Figure 2E,F illustrate that pulmonary rehabilitation induced an increase in the pulmonary levels of the anti-viral cytokines IFN-γ (*p* < 0.0234) and IFN-β (*p* < 0.0010). Additionally, Figure 2G,H demonstrate a notable elevation in the pulmonary levels of the anti-inflammatory cytokines IL-10 (*p* < 0.0001) and IL-1RA (*p* < 0.0001) as a consequence of pulmonary rehabilitation. These findings collectively highlight the substantial systemic anti-inflammatory effects associated with the pulmonary rehabilitation regimen employed in this study.

### 4.4. Pulmonary Rehabilitation Improves Lung Function

**Figure 3:**
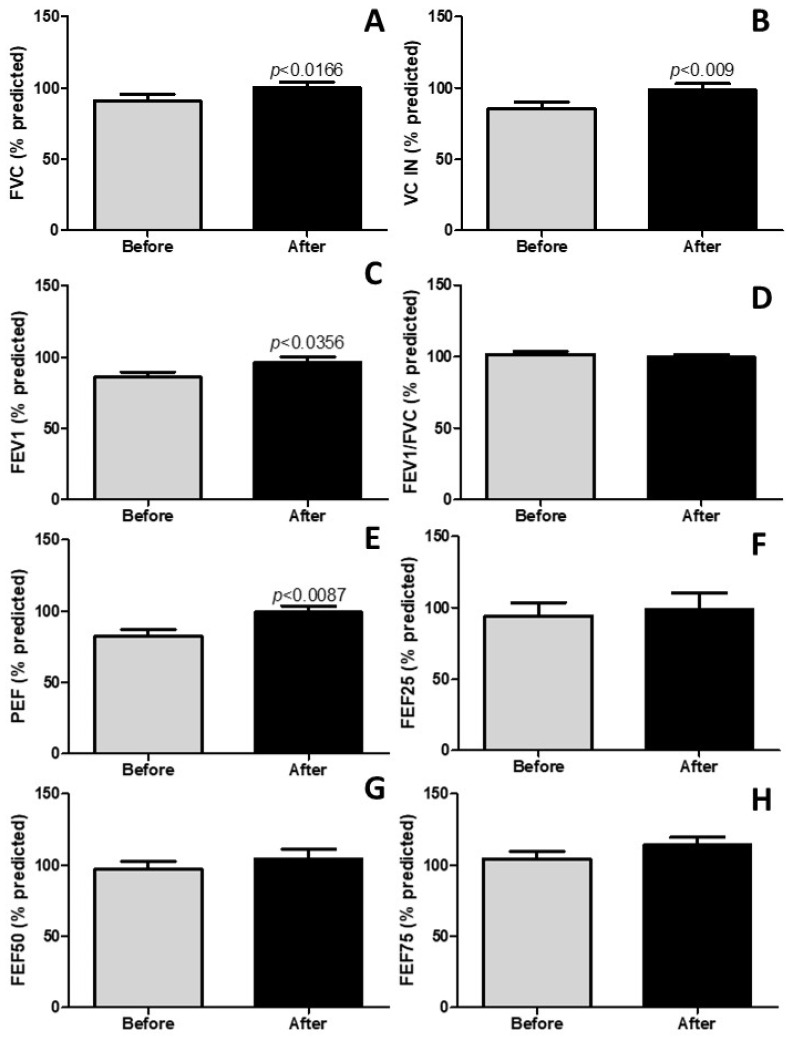
Lung function (spirometry) parameters. (**A**) shows the FVC stands for forced vital capacity. (**B**) shows the VC IN represents vital capacity inspiratory. (**C**) shows the FEV1 denotes forced expiratory volume in the first second. (**D**) shows the FEV1/FVC means a ratio among these two parameters (Tiffeneau–Pinelli index). (**E**) shows the PEF stands peak expiratory flow. FEF represents forced expiratory flow, which is measured at 25% (**F**), 50% (**G**), and 75% (**H**) of FVC. The results are expressed in % of the predicted value.

The findings presented in Figure 3 underscore a significant enhancement in lung function. As illustrated in Figure 3A–C, it becomes apparent that pulmonary rehabilitation resulted in a noteworthy improvement in the forced vital capacity (FVC%) (*p* < 0.0166), ventilatory capacity inspiratory (VC IN%) (*p* < 0.009), and forced expiratory volume in 1 s (FEV1%) (*p* < 0.0356), respectively. Furthermore, pulmonary rehabilitation exhibited a positive impact on peak expiratory flow (PEF%) (*p* < 0.0087). Conversely, Figure 3D,F–H indicate no discernible influence of pulmonary rehabilitation (*p* > 0.05). These collective findings underscore the substantial effects of pulmonary rehabilitation on the improvement of lung function in post-COVID-19 patients.

### 4.5. Pulmonary Rehabilitation Improves Lung Mechanics

**Figure 4:**
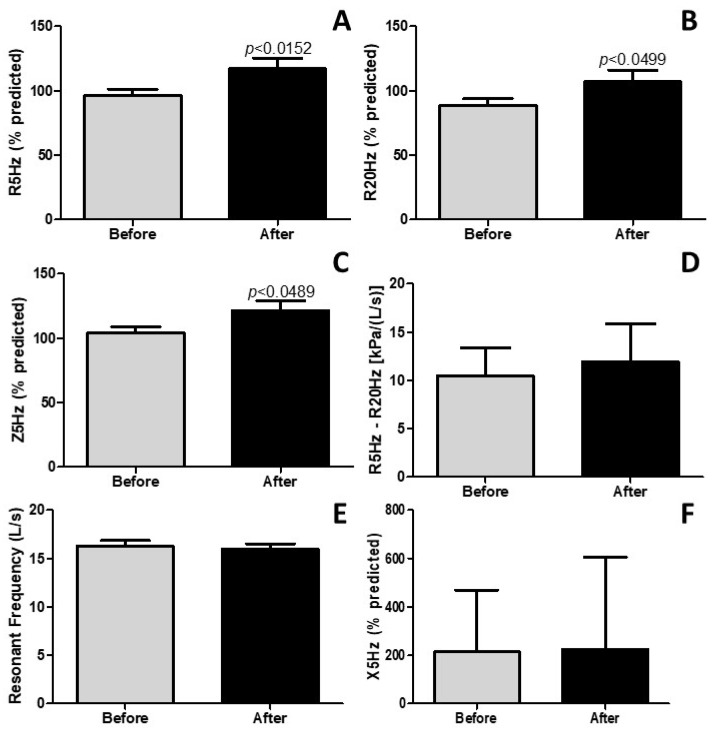
Lung mechanics (impulse oscillometry system—IOS) parameters. R5Hz (**A**) stands resistance of the whole respiratory system. R20Hz (**B**) represents the resistance of the proximal airways. Z5Hz (**C**) denotes the impedance of the respiratory system. R5Hz-R20Hz (**D**) stands for the resistance of distal (small) airways. Resonant frequency (**E**) represents resonant frequency itself. X5Hz (**F**) denotes the reactance of the respiratory system. The results are expressed in % of the predicted value and also in absolute value [kPa/(L/s)].

The results presented in Figure 4 highlight a significant improvement in lung mechanics. As shown in Figure 4A–C, it is evident that pulmonary rehabilitation led to a notable enhancement in the resistance of the entire respiratory system (%) (R5Hz; *p* < 0.0152), proximal airways (%) (R20Hz; *p* < 0.0499), and respiratory system impedance (%) (Z5Hz; *p* < 0.0489), respectively. However, pulmonary rehabilitation did not elicit changes in the resistance of distal (small) airways (%) (R5Hz-R20Hz; *p* > 0.05), resonance frequency (Fres; *p* > 0.05), and reactance (X5Hz; *p* > 0.05). These collective findings underscore the substantial effects of pulmonary rehabilitation on the restoration of lung mechanics in post-COVID-19 patients.

## 5. Discussion

The COVID-19 pandemic, triggered by the highly infectious SARS-CoV-2 virus, has left a profound global footprint, impacted millions of individuals, and yielded a spectrum of clinical manifestations, ranging from mild, to severe, and even fatal outcomes [1,2]. While the primary respiratory effects of COVID-19 have been extensively documented, emerging evidence points to the virus’s potential long-term repercussions on various organ systems [1,2]. This study sought to investigate the lingering effects of COVID-19 and to elucidate the potential benefits of a 12-week pulmonary rehabilitation program for a cohort of moderate and severe post-COVID-19 patients. In summary, pulmonary rehabilitation led to improvements in lung function and mechanics, as well as reductions in both systemic and pulmonary inflammation.

Our results present some evidence of the promising effects of pulmonary rehabilitation in individuals recovering from post-COVID-19. The findings reveal significant enhancements in various clinical parameters, encompassing body weight, BMI, waist circumference, fat mass, and fat-free mass. These improvements underscore the program’s efficacy in addressing some of the physical consequences commonly associated with COVID-19, such as weight gain and alterations in body composition, including the loss of muscle mass and strength [24]. Notably, the present study also highlights that the pulmonary rehabilitation program contributed to improvements in hand grip strength and respiratory muscle strength, further contributing to the overall recovery of individuals post-COVID-19.

Furthermore, it is noteworthy that several post-COVID-19 patients have been reported to experience elevated heart rate and blood pressure, including both systolic and diastolic measurements [25]. In line with these observations, the present study identified favorable changes in resting heart rate and blood pressure among participants, suggesting another advantageous outcome associated with pulmonary rehabilitation. Moreover, resting partial oxygen saturation (SpO_2_%) represents a significant parameter related to the pulmonary sequel of COVID-19 [26]. In this context, our findings revealed a substantial improvement in resting SpO_2_% among our cohort of patients following 12 weeks of pulmonary rehabilitation, further emphasizing the beneficial effects associated with this intervention. Conversely, the bioavailability of fractional exhaled nitric oxide (FeNO) has been reported to be reduced in COVID-19 patients, potentially reflecting impairments in pulmonary circulation [24,27]. Interestingly, the present study demonstrated that the pulmonary rehabilitation program implemented in our study led to a significant increase in SpO_2_%, highlighting its positive impact on pulmonary function.

A noteworthy aspect of this investigation lies in its comprehensive assessment of inflammatory mediators within both breath condensate and serum. Pulmonary rehabilitation yielded a marked reduction in pro-inflammatory cytokines, notably IL-1β and IL-6, both at local and systemic levels. This reduction in inflammatory response assumes paramount significance due to the established association between excessive inflammation and the severity of COVID-19 as well as post-COVID-19 syndrome [28].

Moreover, the rehabilitation regimen engendered an increase in anti-viral cytokines IFN-γ and IFN-β, indicating an enhanced antiviral defense mechanism [29]. Additionally, the observed elevation in anti-inflammatory cytokines IL-10 and IL-1RA suggests a shift in the immune response towards an anti-inflammatory state [30]. Recent evidence underscores the potential utility of IL-10 as an indicator of disease severity and mortality in individuals afflicted by acute or post-acute SARS-CoV-2 infection [30]. In this context, IL-10 may serve as an intrinsic warning signal, released by compromised tissues as a protective mechanism against potentially detrimental hyperinflammatory responses [30]. Similarly, IL-1RA is recognized as a sensitive biomarker associated with disease severity and mortality in COVID-19 patients [31]. It is noteworthy that the pulmonary rehabilitation protocol implemented in this investigation led to a significant elevation in both pulmonary and systemic levels of IL-10 and IL-1RA. This provides compelling evidence of the pronounced anti-inflammatory effects associated with pulmonary rehabilitation.

These findings are in concordance with previous research, which has also demonstrated the robust anti-inflammatory effects of pulmonary rehabilitation in other respiratory conditions, such as asthma [15], COPD [15], and even among obese women [17]. Thus, these collective outcomes underscore the potent anti-inflammatory potential of pulmonary rehabilitation, thereby underscoring its pivotal role in mitigating both pulmonary and systemic manifestations of COVID-19.

The observed amelioration in pulmonary function and mechanics within the scope of this study bears paramount significance. Pulmonary rehabilitation elicited noteworthy enhancements in forced vital capacity (FVC), inspiratory vital capacity (VC IN), forced expiratory volume in one second (FEV1), and peak expiratory flow (PEF), indicative of an improved respiratory function. These observed alterations hold particular relevance in the context of post-COVID-19 individuals, who frequently contend with persistent respiratory symptoms stemming from the compromise of lung function [32]. Furthermore, the favorable impact on lung mechanics, encompassing an improvement in the resistance of both the respiratory system (R5Hz) and proximal airways (R20Hz) (achieving close to 100% of predicted value), serves to underscore the rehabilitative potential in addressing respiratory impairments. On the other hand, the respiratory system impedance (Z5Hz), which was already close to 100% before pulmonary rehabilitation and significantly increased after rehab, indicates an impairment in the respiratory system impedance. In general, an increase in respiratory system impedance (Z5Hz) may result from increased resistance (R5Hz, R20Hz) and reactance (X5Hz) of the respiratory system. However, in the present study there is no increase in resistance of respiratory system (R5Hz) and of proximal airways (R20Hz). On the other hand, the reactance before the pulmonary rehabilitation was around 200% of the predicted value, and the pulmonary rehabilitation did not reduce the reactance (X5Hz). So, this may be a possible explanation to justify why pulmonary rehabilitation did not result in improvements of respiratory system impedance (Z5Hz) and in reactance (X5Hz). These findings resonate with the existing body of literature, which advocates for an expanded availability of pulmonary rehabilitation programs [33].

Our study also underscores the potential role of exercise-based rehabilitation programs in post-COVID-19 care. While the exercise regimens employed in this study were initially developed for conditions such as COPD [15] and asthma [12], they have shown promise in post-COVID-19 patients. Aerobic training on a treadmill, coupled with resistance exercises, resulted in significant improvements in strength and fitness. The progressive nature of the training load allowed for individualized adjustments, ensuring that patients could safely and effectively engage in the program. Additionally, monitoring signs of fatigue, such as SpO_2_% helped prevent overexertion, ensuring the safety of participants during exercise.

The limitations of this study include the absence of a control group, a challenge posed by the ongoing pandemic and ethical considerations. However, the observed changes in clinical, inflammatory, and physiological parameters suggest the beneficial effects of pulmonary rehabilitation. Future research including comparisons with a non-intervention group or other types of rehabilitation with larger cohorts and control groups may provide further insights.

This study emphasizes the clinical significance of early intervention through pulmonary rehabilitation for post-COVID-19 patients, as performed in the present study. Our results demonstrate that pulmonary rehabilitation not only improves lung function but also lung mechanics, as well as systemic and pulmonary inflammation and immune responses. These positive effects may provide benefits to post-COVID-19 patients not only in the acute phase but also in the chronic phase, potentially preventing a poor prognosis. Furthermore, although not tested directly in the present study, it is proven that increases in the pulmonary and systemic levels of anti-viral cytokines (IFN-γ and IFN-β) and anti-inflammatory cytokines (IL-10 and IL-1RA) may prevent re-infections [29,30,31,32,33].

## 6. Conclusions

In conclusion, our study provides initial evidence of the positive impact of a 12-week pulmonary rehabilitation program on moderate and severe post-COVID-19 patients. The program led to improvements in clinical parameters, reduced pulmonary and systemic inflammation, and enhanced lung function and mechanics.

## Figures and Tables

**Table 1:** Clinical and anthropometric characteristics of post-COVID-19 patients after and before rehabilitation.

PARAMETERS	Before	After	*p* Value
Age (years)	45.44 (3.5)	46.52 (3.1)	0.2737
Weight (kg)	74.03 (4.2)	65.89 (6.3)	0.0002
Height (m)	1.68 (4.2)	1.68 (4.6)	0.5086
BMI (kg/m^2^)	30.43 (2.3)	25.88 (3.5)	0.0002
Systolic blood pressure (mmHg)	142.1(1.4)	118.1(1.4)	0.0002
Diastolic blood pressure (mmHg)	95.4 (0.7)	74.4 (0.9)	0.0002
Waist circumference (cm)	92.24 (4.7)	78.85 (1.6)	0.0004
Total leukocytes (cells/mm^3^)	7.96 (2.0)	6.22 (2.7)	0.5940
Basophils (cells/mm^3^)	34.5 (7.0)	26.8 (8.7)	0.0089
Monocytes (cells/mm^3^)	414.5 (4.1)	218.42 (3.2)	0.0026
Eosinophils (cells/mm^3^)	158.22 (2.6)	146.42 (2.8)	0.7044
Lymphocytes (cells/mm^3^)	3.5 (2.4)	2.46 (2.8)	0.2544
Neutrophils (cells/mm^3^)	4.25 (2.3)	2.52 (2.3)	0.0004
NLR (cells/mm^3^)	10.31 (0.54)	1.88 (0.21)	0.0001
Resting heart rate (bpm)	112.2 (3.2)	85.6 (2.5)	0.0001
Fat mass (%)	42.57 (4.2)	35.89 (2.4)	0.0001
Fat free mass (%)	62.35 (3.5)	64.48 (4.6)	0.0002
Resting SpO_2_%	92.05 (0.6)	97.25 (0.8)	0.0001
FeNO (ppb)	4.07 (1.2)	10.02 (1.2)	0.0001
Sex (M/F) ^#^	14/19		
Grip strength (Kg/force)	40 (5.7)	65 (4.6)	0.0005
MIP (cmH_2_O)	−40.35 (8.9)	−68.35 (7.6)	0.0001
MEP (cmH_2_O)	70.06 (6.3)	95.3(5.8)	0.0001

Clinical and anthropometric characteristics of COVID-19 rehabilitation (n = 33). Mean (± standard deviation), median (interquartile interval). BMI: body mass index; SpO_2_%: pulp oxygenation level, NLR: neutrophil lymphocyte ratio. FeNO: Fractional exhaled nitric oxide. PPB: parts per billion. ^#^ Data in absolute numbers (n). MIP: maximal inspiratory pressure, MEP: maximal expiratory pressure.

## Data Availability

All raw data will be freely available under a reasonable request directly to the corresponding author.
